# A programmable microfluidic platform to monitor calcium dynamics in microglia during inflammation

**DOI:** 10.1038/s41378-024-00733-1

**Published:** 2024-08-01

**Authors:** Adam Shebindu, Durga Kaveti, Linda Umutoni, Gia Kirk, Michael D. Burton, Caroline N. Jones

**Affiliations:** 1 Department of Bioengineering, University of Texas at Dallas, Richardson, TX 75080 USA; 2 Department of Biomedical Engineering, UT Southwestern Medical Center, Dallas, TX 75390 USA; 3 Department of Neuroscience, University of Texas at Dallas, Richardson, TX 75080 USA

**Keywords:** Engineering, Nanoscience and technology

## Abstract

Neuroinflammation is characterized by the elevation of cytokines and adenosine triphosphate (ATP), which in turn activates microglia. These immunoregulatory molecules typically form gradients in vivo, which significantly influence microglial behaviors such as increasing calcium signaling, migration, phagocytosis, and cytokine secretion. Quantifying microglial calcium signaling in the context of inflammation holds the potential for developing precise therapeutic strategies for neurological diseases. However, the current calcium imaging systems are technically challenging to operate, necessitate large volumes of expensive reagents and cells, and model immunoregulatory molecules as uniform concentrations, failing to accurately replicate the in vivo microenvironment. In this study, we introduce a novel calcium monitoring micro-total analysis system (CAM-μTAS) designed to quantify calcium dynamics in microglia (BV2 cells) within defined cytokine gradients. Leveraging programmable pneumatically actuated lifting gate microvalve arrays and a Quake valve, CAM-μTAS delivers cytokine gradients to microglia, mimicking neuroinflammation. Our device automates sample handling and cell culture, enabling rapid media changes in just 1.5 s, thus streamlining the experimental workflow. By analyzing BV2 calcium transient latency to peak, we demonstrate location-dependent microglial activation patterns based on cytokine and ATP gradients, offering insights contrasting those of non-gradient-based perfusion systems. By harnessing advancements in microsystem technology to quantify calcium dynamics, we can construct simplified human models of neurological disorders, unravel the intricate mechanisms of cell-cell signaling, and conduct robust evaluations of novel therapeutics.

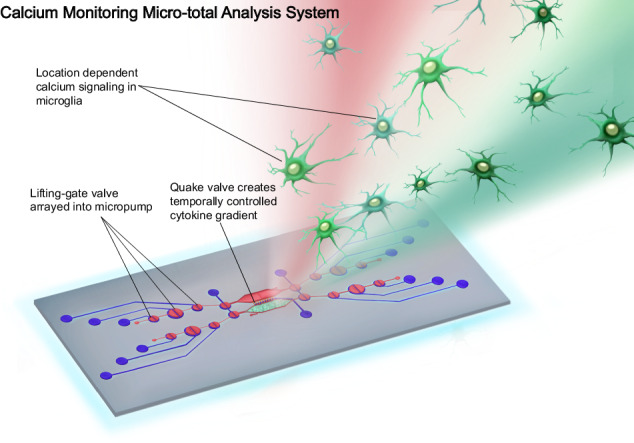

## Introduction

Microglia are central nervous system (CNS)-specific immune cells that respond to both immunological^1^ and neuronal^2,3^ signals. They are a subset of glia cells, which play an important role in maintaining homeostasis in the CNS^4^. Abnormal calcium signaling in glia cells has been associated with neuroinflammation^5^ which underlies several neurodegenerative diseases, including Alzheimer’s disease^6^, Parkinson’s disease^7^, multiple sclerosis^8^, pain^9^, and sepsis^10^. Recent studies in mice have demonstrated the response of microglia to neuronal activity through spontaneous calcium dynamics^2,11^. Moreover, calcium signaling is involved in mediating intercellular communication among microglia, as well as between microglia and other cell types, such as neurons and astrocytes during microglia activation^2,12^. When cytokines engage their respective receptors, they stimulate calcium-dependent second messenger systems, mitochondrial and endoplasmic calcium store release, and ATP-induced calcium transients^3^. Therefore, studying calcium dynamics aids in unraveling the dynamics of these communication networks and their impact on brain function and is valuable for identifying novel therapeutic strategies and assessing the efficacy of drug interventions.

**Fig. 1:**
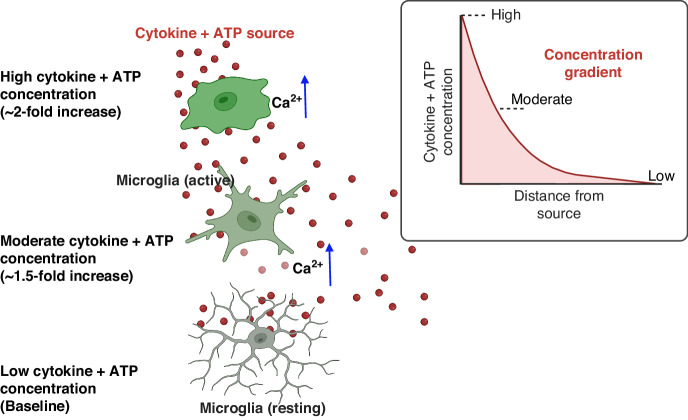
Location-dependent microglia response to cytokine gradients. In vivo, microglia respond to neuronal (ATP) and immune stimuli (cytokines) through a concentration gradient. Cells closer to the area of inflammation are activated first due to a high cytokines concentration. As the signal propagates across the CNS, the concentration becomes moderate before completely diffusing

Current in vitro calcium dynamics studies are done using a perfusion system that takes hours to set-up, use a high volume of reagents, requires millions of cells making it challenging to study human cells, and delivers treatment to cells through a uniform concentration of inflammatory signals^11,13^. However, microglia sense and respond to inflammation in the human CNS through a concentration gradient of inflammatory signals such as cytokines, chemokines, or adenosine triphosphate (ATP)^14^^,^^15^. Therefore, creating a concentration gradient during the delivery of treatment to the cells is biomimetic, which increases the effectiveness of imaging calcium dynamics^12,16,17^(Fig. 1). The requirement to generate a gradient strengthens the demand for new microfluidics biomimetic tools that are efficient in terms of set-up time, reduction in cell sample size, and delivery of treatments to the cells.

Microfluidic systems are miniaturized and can be automated to deliver treatment to cells and generate a concentration gradient^17^. These systems have recently been used for calcium imaging in different in-vitro models including osteoblasts^18^, neuronal communication^19–21^, and astrocyte activation^22^. Additionally, Chokshi et al. introduced an automated microfluidic technology for the in vivo study of calcium dynamics in *Caenorhabditis elegans*. This device enabled the processing of thousands of worms by immobilizing them, delivering a chemical odor to their nose touch, and recording the calcium transient data from single neurons while eliminating manual interactions with the device^23^. However, the presented technologies have been limited by either the lack of automation during the treatment delivery or the creation of gradients to make the system biomimetic.

During the last two decades, microfluidic systems have been automated by incorporating fluidic control mechanisms, including electrowetting^24^, acoustic^25^, and pneumatic actuations^26–29^. Among these actuation mechanisms, pneumatically actuated microvalves and micropumps have gained prominence due to their ease of fabrication, accuracy in volume control, high scalability, and simple integration with downstream systems. Microvalves and micropumps have been incorporated into microfluidic devices for fluidic control in several applications, including pathogen biosensing^30–32^, molecular preconcentration^33^, immunoassays^34^, and high throughput cellular analysis^35,36^. Pneumatically actuated microvalves are divided into Quake valves and lifting-gate valves which are used separately based on the application. Lifting-gate valves are highly efficient with pumping efficiencies reported of up to 86%^27^, whereas the Quake valve uses a mechanical force to pinch a fluidic membrane and interrupt the flow^37,38^. The Quake valve can be used as a blocking valve to create flow separation in channels of a microfluidic device^39,40^. Both valve types use a monolithic, flexible PDMS membrane for actuation. However, they have several limitations. For instance, lifting gate valves produce backflow during actuation, and Quake valves have poor resolution for flow metering. Table 1 summarizes the advantages and limitations of these valve systems. Despite offering the ability to pump efficiently while blocking fluid flow at specific positions, no system combining Quake valves and lifting-gate valves has been introduced.

**Table 1:** Comparison of pneumatically actuated microsystems

Valve type	Advantages	Limitations	Applications
**Lifting-gate valve (normally closed, actuate to open)**	High pumping efficiency Used in series to form micropumps	Need for valve passivation to avoid them becoming permanently closed Pumping backflow causes unsteady laminar flow	Precise metering of reagents^33^ Rapid biosensing and point-of-care diagnostics^30^
**Quake valve (normally opened, actuate to close)**	Used to interrupt flow without causing unsteady laminar flow Used to create a concentration gradient	Poor resolution for flow metering The height and the width of the valve cannot be designed independently	Immunoassays^50^ Cell sorting^51^
**System combination of lifting gate and quake valves**	Leveraged the pumping capabilities of normally closed valves for high-efficiency fluidic manipulations Uses normally opened valves to block flow at a targeted location to create isolated chambers Allows the formation of a concentration gradient in channels	Fabrication challenges due to non-compatibility of photoresists and developers for normally closed and normally opened valves	Drug screening to cells Cell co-culture Cell migration to a gradient

We present a calcium monitoring micro-total analysis system (CAM-μTAS) that combines both the Quake and lifting gate valves. The integration of the pumping capabilities of lifting gate microvalves with the flow interrupting capabilities of the Quake valve enables quantitative analysis of calcium dynamics in microglial cells after exposure to a gradient of cytokines with temporal control. We validated this system and successfully automated calcium imaging of microglia during a dosed treatment of an “inflammatory soup” consisting of interleukin-1β, (IL-1β), interleukin-6 (IL-6), tumor necrosis factor-α (TNF-α), and adenosine triphosphate (ATP). These pro-inflammatory cytokines are upregulated at the site of inflammation in the CNS^41^ and ATP, a nucleotide molecule, is involved in cytokine recruitment during inflammatory cascades and facilitates phagocytosis in the central nervous system (CNS).^42^. This system automatically changed cell culture media with high efficiency, prepared cells for calcium imaging by on-chip calcium indicator incubation and exposed the cells to a concentration gradient of IL-1β, IL-6, TNF-α, and ATP. The CAM-μTAS also includes a pneumatically controlled microfluidic flow rectifier that enables zero backflow in all operations using the principle of an electrical transistor^43^. Eliminating backflow and ensuring steady flow allows the stabilization of cells during continuous pumping cycles of the lifting gate valves. As a result, the developed CAM-μTAS can be utilized in different cell models for calcium imaging.

## Materials and methods

### Materials and equipment

The CAM-μTAS was fabricated by photolithography followed by soft lithography. To create the mold, we used SU-8 50 (Kayaku Advanced Materials, Westborough, MA), a negative photoresist, and AZ-12XT-20PL-10 (Microchemical GmbH, Germany), a positive photoresist, and silicon wafers (University Wafer, Inc., Boston, MA). For soft lithography, we used polydimethylsiloxane (PDMS) (Dow Corning, Midland, MI). To control the microvalve system, a control system was made using a series of SMC 3 solenoid valves (Steven Engineering, San Francisco, CA), ULN2803 switchboards (Tempero Systems, Southport, Australia), and data acquisition (DAQs) devices (National Instruments, Austin, TX). The DAQs were connected to a laptop and controlled using a custom-made NI-LabVIEW program. All images were acquired by an inverted microscope (Nikon Ti-E, NY) equipped with a CCD camera (EMCCD, Andor).

### Cell culture

To perform calcium dynamics on a chip, we used BV2 cells- a C57BL/6 murine microglia cell line gifted by Malu G. Tansey (The University of Florida, Gainesville, FL). Upon receival, BV2 cells were grown in a medium constituted by 41.5% high glucose Dulbecco’s Modified Eagle’s Medium (DMEM, Gibco, Waltham, MA), 41.5% Opti-MEM (Gibco, Waltham, MA), 15% fetal bovine serum (FBS, Gibco, Waltham, MA), and 2% penicillin streptomycin (Gibco, Waltham, MA). BV2 cells were seeded in 25-cm^2^ flasks at a density of 2 × 10^5^ cells/mL and placed in a humidified atmosphere incubator containing 5% CO_2_ at 37 °C. The cell culture media was exchanged as needed (minimally twice a week) to supplement the cell line with fresh nutrients.

### Calcium imaging using a perfusion system

Calcium imaging was performed as previously described by Li et al^13^. Briefly, BV2 cells were plated on 0.1% poly-l-lysine coated glass bottom single well plates at a 2 × 10^6^ density and placed in a humidified atmosphere incubator containing 5% CO_2_ at 37 °C overnight. The next day, cells were incubated with calbryte 520-AM [4 µM] (AAT Bioquest, Pleasanton, CA) at 37 °C for 30 min. Following the incubation, cells were washed with an aqueous normal bath comprised of the following: 13.5 mM NaCl, 5 mM KCl, 10 mM HEPES, 2 mM CaCl_2_, 1 mM MgCl_2_, 10 mM glucose, titrated to pH 7.4 ± 0.5. A custom-made perfusion system was used to deliver an inflammatory soup constituted of IL-1β [10 ng/mL] (R&D System, Minneapolis, MN), IL-6 [50 ng/mL] (R&D System, Minneapolis, MN), TNF-α [100 ng/mL] (R&D System, Minneapolis, MN), and ATP [200 µM] (Sigma-Aldrich, St. Louis, MO), at a flow rate of 500 µL per second. While perfusing, calcium dynamics images were recorded using an Olympus IX73 inverted microscope (40X magnification, 100 ms exposure time) as shown in SI Fig. 2A.

### Fabrication of the calcium monitoring micro-total analysis system (CAM-μTAS)

**Fig. 2:**
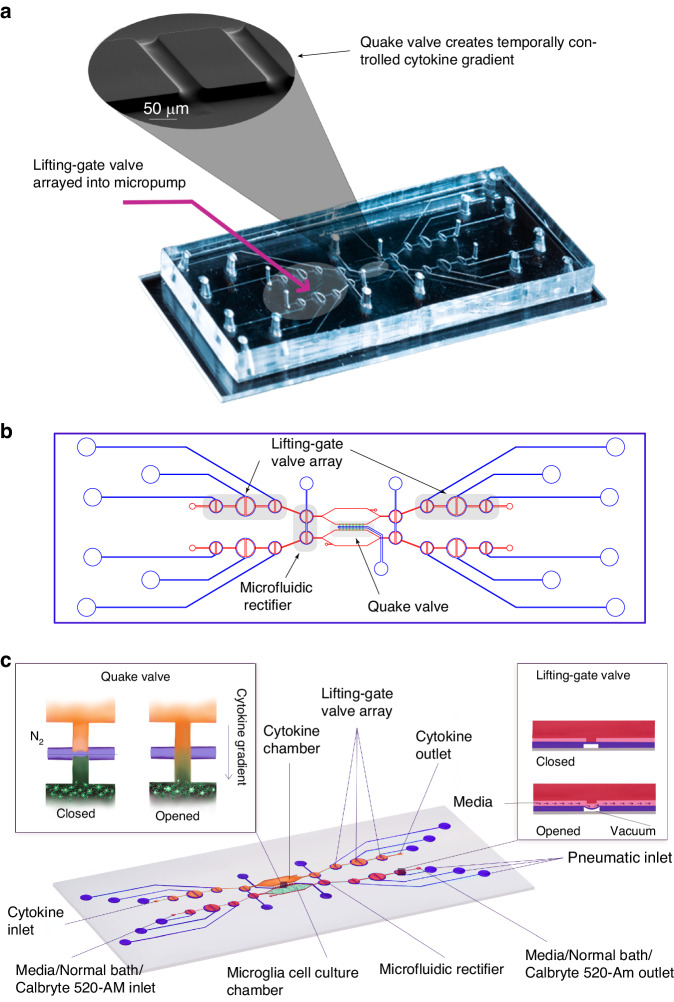
Calcium monitoring micro-total analysis system (CAM-μTAS) to quantify calcium dynamics in microglial cells. **a** A fabricated CAM-μTAS, including the lifting gate valve and a scan electron microscopy image of the Quake valve. **b** CAD design of the CAM-μTAS, including lifting gate valves, microfluidic rectifiers, and a quake valve. **c** A feature illustration of the CAM-μTAS. The device combines a series of lifting gate valves into a micropump to automatically and precisely meter cell culture media changes, load calbryte 520-Am, and eliminate backflow. The CAM-μTAS leverages the flow-blocking capability of the Quake valve to generate a concentration gradient, which enables a biomimetic delivery of inflammatory signals to the cells

The CAM-μTAS illustrated in Fig. 2 consisted of a microfluidic layer which included the microglia cell culture and cytokine chambers, a pneumatic control layer, and a featureless glass wafer. The pneumatic layer was fabricated using standard photolithography techniques with SU-8 50 to achieve a feature height of 80 µm. A multilayer photolithography technique was used to fabricate the fluidic layer and obtain 40 µm feature height for the lifting gate valves and the chambers, and 7 µm for the Quake valves. First, SU-8 50 was spin-coated on a silicon wafer at 3000 rpm for the fabrication of the lifting gate valves and the chambers. To fabricate the Quake valve after developing the SU-8, the wafer was primed with hexamethyldisilazane (HMDS) for adhesion promotion, then spin-coated with AZ-12XT-20PL-10, a positive photoresist at 3500 rpm. The photoresist was cured for 3 min at 115 °C then exposed to UV at a dose of 450 mJ/cm^2^ before baking it at 100 °C for 1 min and developing it by submersion in AZ 300MIF for 3 min. The Quake valve requires semi-circular channels for operation^37^. To achieve this, the wafer was placed on a hot plate at 135 °C to allow the reflow of AZ-12XT-20PL-10. This process is summarized in SI Fig. 1. After coating both the fluidic and pneumatic layers with parylene-C (Specialty Coating Inc.) as an anti-sticking agent, a 10:1 ratio of PDMS (base elastomer: curing agent) was poured onto the molds to obtain replicas^33^. For the pneumatic layer, uncured PDMS was spin-coated on the mold at 915 rpm to obtain a 100-μm-thick PDMS membrane and for the fluidic layer, PDMS was poured on the mold. Both were cured at 65 °C in an oven overnight. Holes were punched in the PDMS replica with a biopsy punch for fluidic inlets and outlets, and then the fluidic layer was aligned and permanently bonded to the pneumatic layer using air plasma activation (Plasma Etch Inc.) at 350 mTorr chamber pressure and 90 W power for 3 min to form the CAM-μTAS. Lastly, holes were punched in the CAM-μTAS for pneumatic connection wells and the CAM-μTAS was bonded to a single well glass bottom plate (MatTek) after air plasma activation and placed on a hot plate at 80 °C for 15 min.

### Chip operation and flow optimization around cells

The CAM-μTAS was optimized for flow rate by applying vacuum for opening at −85 kPa and varying the closing pressures at 15, 30, and 45 kPa to the pneumatic lines by the solenoid controller. The volume pumped per cycle is a function of closing pressure and actuation time^27^. SI video 1 summarizes the pumping mechanism used for the device's operation. After flow optimization, a closing pressure of 45 kPa and an actuation time of 500 ms were selected because this condition yielded the highest flowrate (355 nL/cycle) at the lowest actuation time (500 ms). Prior to operating the CAM-μTAS, all microvalves were opened and the chambers were filled with 0.1% poly-l-lysine for 2 h to functionalize the PDMS surface and improve cell adhesion. After 2 h, poly-l-lysine was washed three times by pumping in sterile DI water before adding cell culture media. Microglia (BV2) cells were added to the microglia cell culture chamber at 2 × 10^6^ cells/mL, and the chip was removed from the controller and placed in a humidified incubator with a 5% CO_2_ atmosphere at 37 °C for 24 h to allow the cells to adhere. The next day, the chip was installed on the controller, and 25 cycles were pumped to test the rate of detachment of cells from the surface of the chamber. This process was repeated under different surface treatments, including 0.1% poly-l-lysine, and 0.1% poly-l-lysine mixed with fibronectin [11 µg/mL]. While pumping, time-lapse images were taken at an interval of 1 s for 3 min at 20X magnification to observe the rate of cell detachment. This rate was quantified in terms of the percentage of cells removed from the field of view after the 25 pumping cycles.

### Automated calcium indicator incubation

To perform calcium imaging, cells are incubated with calbryte 520-AM. A 4 µM solution of calbryte 520-AM was prepared in an aqueous normal bath comprised of the following: 13.5 mM NaCl, 5 mM KCl, 10 mM HEPES, 2 mM CaCl_2_, 1 mM MgCl_2_, 10 mM glucose, titrated to pH 7.4 ± 0.5. The normal bath was adjusted to an osmotic pressure of 300 ± 5 mOsm. Three cycles of calbryte 520-AM were pumped into the microglia cell culture chamber and the cells were incubated at 37 °C for 30 min. Time-lapse images were taken using the Nikon Ti-2 fluorescent microscope with a biochamber (FITC, 20X magnification).

### On-chip microglia calcium imaging

To demonstrate the utility of the CAM-μTAS, the microglia cell culture chamber was primed with 0.1% poly-l-lysine for 2 h at room temperature. After washing the poly-l-lysine by pumping three cycles of sterile DI water, 400 nL of BV2 cells suspended in media at a density of 1.98 × 10^6^ cells/mL (~800 cells) were loaded into the microglia cell culture chamber. The CAM-μTAS was removed from the controller and placed in a humidified incubator with 95% air and 5% CO_2_ atmosphere at 37 °C for 24 h to allow the BV2 cells to adhere. The next day, the media was replaced by a normal bath solution described above. Calbryte 520-AM was pumped into the system and then completely washed out after 30 min. The CAM-μTAS was placed on the Nikon Ti-2 fluorescent microscope mounted with an incubator at 37 °C. In the adjacent chamber, a solution diluted in normal bath and containing IL-1β [10 ng/mL], IL-6 [50 ng/mL], TNF-α [100 ng/mL], and ATP [200 µM], was added while the Quake valve was closed. Once the treatment was in place and the cells were fluorescent with calbryte 520-AM, the Quake valve was opened, and a gradient was allowed to form for 10 min. We repeated this experiment in three devices.

### Statistical analysis

Statistical analysis was performed using Prism-GraphPad 8. Data from at least three experiments were analyzed and presented as mean ± standard deviation. For a given experiment, each condition was tested in triplicate. One-way ANOVA with a statistical significance level of 0.05 was used to determine the difference in chip surface treatment for cell adhesion.

### Image analyses

Image analyses were performed using Nikon Elements AR 5.41.02 and data were plotted using Prism-GraphPad (San Diego, California). For calcium dynamics, data are expressed in ΔF/F ratio where F is the intensity at time *t* = 0 s while ΔF is the difference in intensity at time *t* = 0 s, and at the time of cell response.

## Results

### Combining lifting gate valves and Quake valve to create the CAM-μTAS

The lifting gate valves, and the Quake valve have distinct fabrication techniques which require negative and positive photoresist, respectively. To incorporate the two valve systems on the same device, the fluidic layer was fabricated using a photomask printed with the lifting gate valve circuit and another photomask printed with the Quake valve. During fabrication, the lifting gate valves are fabricated first because positive photoresists are not compatible with SU-8 developers. The Quake valve layer is aligned to the lifting gate circuit as shown in SI Fig. 1. The components of the circuit are summarized in Table 2. To operate the Quake valve, fluidic channels must have a semi-circular roof to allow the membrane to deflect. We fabricated the Quake valve channels with a height of 7 µm and a width of 50 µm to eliminate the dependence of actuation pressure on the depth of the fluidic channel (SI Fig. 1). The fabricated device was mounted to a control system as illustrated in SI Fig. 2B and used the push-up method for pneumatic actuation^38^. Mounted to a pneumatically controlled manifold, the lifting gate valve system operates as an alternating flow generator while the Quake valve operates as a programmed switch (Table 2).

**Table 2:** Microfluidic circuit components (Scale bar: 1 mm)

Component	Image	Function	Circuit symbol
Lifting-gate valve Micropump		To pump reagents including cell culture media, normal bath, or calcium indicator to cells	
Quake valve		To create a chemical gradient during the delivery of cytokines cells	
Microfluidic rectifier (diodic valve)		Ensure backflow elimination and stabilization of flow within the cell culture chamber	
Gate valve		Allow separation of downstream pump from the cell culture and cytokine chambers	
Cytokine and cell culture chambers		Storage of cytokines and cells	
CAM-μTAS		Fully automated microfluidic system to monitor calcium dynamics in microglia following a cytokines concentration gradient-based delivery	

**Fig. 3:**
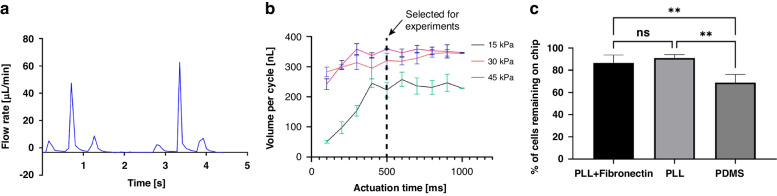
Backflow elimination, and flow optimization for cell adhesion in different surface treatments. **a** Volumetric profile of the flow rate recorded during actuation which shows complete elimination of backflow during actuation. Each peak represents a valve. The high peaks are representative of the pumping valve (middle lifting gate valve) which is double the diameter of the two small gate valves (low peaks). A series of three peaks represent a full actuation cycle **b** Volume pumped per cycle as a function of actuation time and closing pressure of the microvalve. The valve actuation vacuum was −85 kPa, and the pressure varied from 0 to 45 kPa. For the experiment, an actuation time of 500 ms was selected at 45 kPa closing pressure. **c** The percentage of cells retained on the device under 45 kPa valve closing pressure and 500 ms actuation time for different PDMS surface treatments. (Ordinary one-way ANOVA; *n* = 4, **p < 0.05, no significant difference)

Lifting-gate valves have been reported to generate backflow during continuous pumping processes^43^. This continuous instability caused by backflow in the microglia cell chamber can lead to cell detachment or death. To address this, the CAM-μTAS included a pneumatically controlled microfluidic flow rectifier to regulate and reduce the flow rate while pumping around cells while also playing the role of a diode to eliminate backflow (SI Fig. 3). Therefore, prior to the operation of the CAM-μTAS we evaluated the effectiveness of the microfluidic flow rectifier by recording the flow profile during actuation. As seen in Fig. 3a, the positive peaks from the actuation of the lifting gate valves indicate that there is no negative flow. The CAM-μTAS eliminated backflow in the cytokine and microglia cell culture chambers.

### Flow characterization and cell retention

The CAM-μTAS consisted of twelve lifting gate valves, four flow rectifying valves, and one Quake valve to allow access to two fluid inlets and two outlets to the system. The lifting gate valves were arranged in groups of three to include two gate valves with a diameter of 1 mm and one central pumping valve with a diameter of 1.5 mm. The valves were connected by 50 µm wide channels leading to the 4 × 2 mm microglia cell culture and cytokine chambers. The 1.5 mm diameter and the 80 µm pneumatic height of the pumping valve were selected to ensure a maximum deflection of the pneumatic membrane during actuation. The arrangement of the lifting-gate valves in a series of three effectively created micropumps, which were used to transfer cell culture medium, normal bath solutions, calcium indicators to the microglia cell culture chamber, and cytokines to the cytokine chamber.

The pumping flow rate was measured and expressed in terms of volume dispensed per pumping cycle as a function of closing pressure and actuation time. As previously observed^27^, at fixed actuation time, the volume flow rate increased as a function of closing pressure. However, the pumping valve has a maximum membrane deflection height. Therefore, as the actuation time increases, the valve reaches its maximum holding capacity (Fig. 3b). The maximum volume pumped per cycle was reached at 45 kPa closing pressure and 500 ms actuation time. This condition was selected for all the downstream experiments. Under this condition, the lifting gate valve system yielded a pumping efficiency of 73.3% calculated from the theoretical pumping valve capacity.

Microglia are adherent cells that attach to the surface on which they are seeded^44^. However, the adhesion of cells on PDMS surface has been shown to be weak in general^45^. Therefore, the surface requires functionalization to ensure that cells are retained on the surface. We used two different surface treatments, 0.1% poly-l-lysine and 0.1% poly-l-lysine reinforced with fibronectin [11 µg/mL] and tested the cell retention rate against the flow rate at 45 kPa and 500 ms actuation time. We found no significant difference between treating the surface with only poly-l-lysine and poly-l-lysine reinforced with fibronectin (Fig. 3c). Therefore, poly-l-lysine was selected as the surface treatment agent for all the subsequent experiments because it had the lowest average cell detachment.

### Calcium dynamics of microglia on the CAM-μTAS

**Fig. 4:**
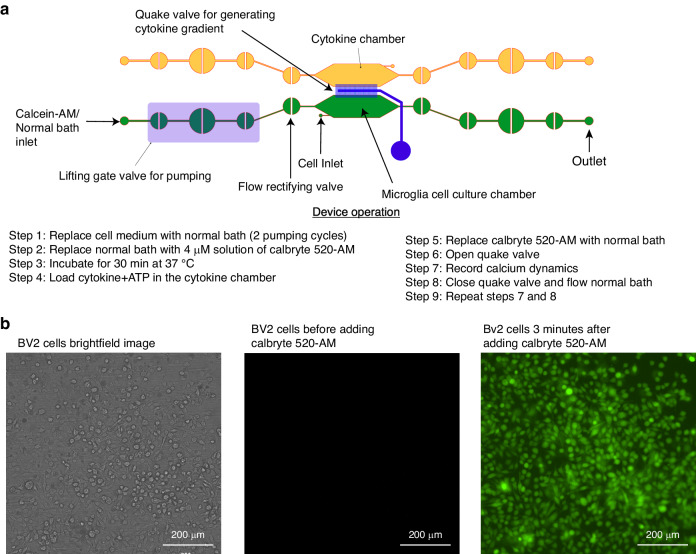
Device operation and calcium indicator loading. **a** The process of actuating the lifting gate valves in series to create a micropump following a continuous flow regime in order to load calbryte 520-AM/normal bath/cell medium to the microglia cell culture chamber and cytokines+ATP to the cytokine chamber. **b** Microglia cells before and after calbryte 520-AM incubation. Miniaturization accelerated the incubation process allowing calbryte 520-AM to penetrate the cells in 30 min. (20X magnification)

**Fig. 5:**
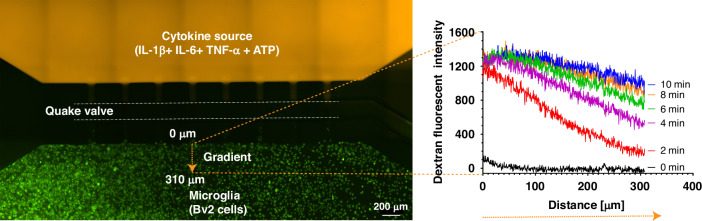
Controlled cytokine gradient. A concentration gradient was generated to deliver pro-inflammatory cytokines (IL-1β [10 ng/mL], IL-6 [10 ng/mL], TNF-α [5 ng/mL], and ATP [200 μM]) to the cells. As seen in the above fluorescence plot, the decreasing concentration of the signal from the signal chamber to the cell culture chamber makes the CAM-μTAS biomimetic. Dextran was added to the cytokine solution for fluorescent imaging purposes

The Quake valve incorporated in the CAM- μTAS played two roles. On one hand, it allows the compartmentalization of the microglia cell culture chamber and the cytokine chamber. On the other hand, it controlled the delivery of treatments to cells through a concentration gradient. The Quake valve enabled us to model inflammation by creating a user-controlled gradient of inflammatory signals toward the microglial cell culture chamber. These signals included IL-1β [10 ng/mL], IL-6 [50 ng/mL], TNF-α [100 ng/mL], and ATP [200 µM]^46^. The signal solution was pumped into the cytokine chamber through the automated lifting gate valve system (Fig. 4a). Simultaneously, Calbryte 520-AM was pumped into the microglia cell culture chamber as a calcium indicator. After 30 min, the cells were fluorescent, as shown in Fig. 4b. Upon opening the Quake valve the gradients reached the BV2 cells. Figure 5 illustrates the progression of the gradient over time as recorded on NI element AR using the TRITC channel of the Nikon-Ti fluorescent microscope. As the gradient formed and the cytokine + ATP treatment was being delivered to the cells, time-lapse images were recorded at 1 s intervals for 10 min starting from the time when the Quake valve was opened.

**Fig. 6:**
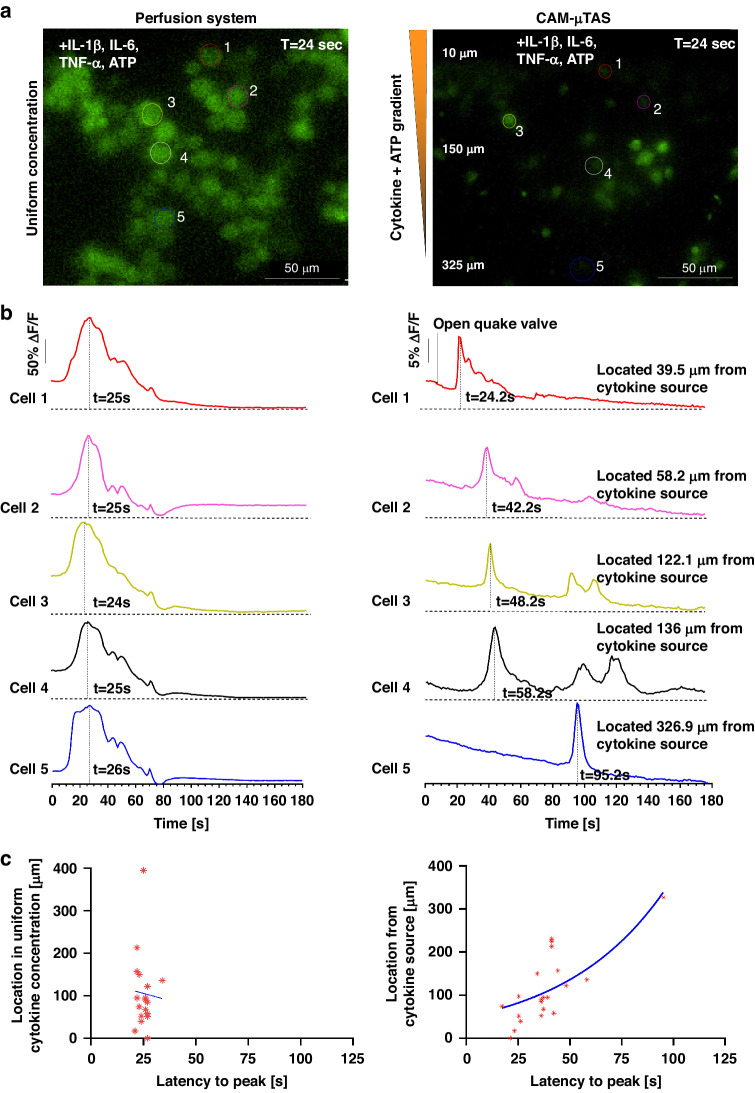
Pro-inflammatory cytokines (IL-1β, IL-6, TNF-α, and ATP) induced a [Ca^2+^] transient elevation in microglia. **a** 40x Fluorescent FITC calcium image of BV2 cells at the peak calcium transient intensity using the perfusion system and the CAM-μTAS. 45% of sampled cells showed calcium transient activity **b** Time series of the fluorescence intensities of responsive cells during the application of IL-1β [10 ng/mL], IL-6 [10 ng/mL], TNF-α [100 ng/mL], and ATP [200 μM] using the perfusion system and using the CAM-μTAS. On the CAM-μTAS, microglia response to the gradient is location-dependent (the locations displayed are measured away from the cytokine source). **c** Location vs. latency to peak (the time it takes for the signal to reach its maximum strength after being initiated) of cells using the perfusion system and using the CAM-μTAS. There is no correlation between location and latency to peak in the perfusion system (*r* = −0.046), but the CAM-μTAS showed a strong correlation (*r* = 0.76) (*n* = 3 devices)

Using the CAM- μTAS we were able to quantify the calcium dynamics in single microglia and observed that 45% of sampled cells showed calcium transient activity (*n* = 43) whereas using the perfusion system the number was 42% (*n* = 54). Conventional cell calcium dynamics imaging uses a perfusion system, which operates with ON/OFF valves to deliver treatment to cells in a stepwise manner as shown in SI Fig. 2c. However, the CAM- μTAS delivers the treatment following a concentration gradient. In the traditional perfusion system, cells responded at the same time (25 ± 2 s) after perfusing the well plate as shown in Fig. 6a, b. However, in the CAM- μTAS, cells responded following a concentration gradient of IL-1β, IL-6, TNF-α, and ATP. Cells closer (<10 μm) to the source started to respond 20 ± 3 s after opening the Quake valve as shown in SI Video 2. As the treatment reached the cells downstream (10–325 μm), there was a location-dependent response with a positive correlation (*r* = 0.76) as shown in Fig. 6c. However, in the perfusion system, all the responsive cells responded at a relatively fixed time with no location dependence (*r* = −0.048). As shown in Fig. 6b, we observed a tenfold decrease in the signal peak intensity of the calcium transient signal using the CAM- μTAS. This is because, unlike the perfusion system which delivers uniform high concentrations of IL-1β, IL-6, TNF-α, and ATP to cells, as shown in SI Fig. 2c, the CAM- μTAS creates a gradient. Therefore, at the time of cell response, the concentrations of IL-1β, IL-6, TNF-α, and ATP are lower than the source concentrations as shown in Fig. 5.

## Discussion

Traditionally, intracellular calcium imaging has been studied using a perfusion system such as the one shown in SI Fig. 2a. Although this system is functional and offers high temporal resolution, it uses a high volume of reagents and a large sample size^13^. In brain science, it is often difficult to obtain such large sample sizes. Moreover, the perfusion system also fails to create a concentration gradient during the delivery of treatments which makes it less biomimetic. In microfluidics, however, cell calcium imaging is performed by using manually operated devices that lack a concentration gradient^47,48^. We contribute to previous efforts by adding an automatically actuated pneumatic microvalve system which combines the lifting gate valves and a Quake valve to generate a concentration gradient as shown in Fig. 1 and set up in SI Fig. 2b. Lifting-gate valves have been previously used for high throughput biochemical processing with reported efficiencies as high as 86%^27,33^. To our knowledge, however, these valves have not been used to automate cell culture mainly because the actuation of lifting gate valves is followed by backflow which can cause cell death. The CAM-μTAS addresses this issue by incorporating a microfluidic flow rectifying valve^43^ which eliminates backflow at the inlet of the cell culture chamber as seen in SI Fig. 3. Adding automation to cell culture enabled us to accelerate sample handling steps including media changes, calcium indicator incubation, and delivery of treatment to cells. These steps normally take several minutes to complete. However, with the CAM-μTAS we achieved them in seconds. For example, to change the medium in the cell microglia cell culture chamber at a 500 ms actuation time (Fig. 3b) only took 1.5 s in total.

Incorporating Quake valves and lifting gate valves on the same device adds complexity to the fabrication process. However, replacing the Quake valve used in the CAM-μTAS with a series of lifting gate valves introduces a significant amount of dead volume which is not suitable for controlling the chemical gradient during the delivery of the treatment to the cells. Therefore, separating the microglia cell culture chamber and the cytokine chamber with a Quake valve not only creates a physical separation between the chambers, but also provides the formation of a user-controlled cytokine gradient. The user can actuate the valve at any time or program the actuation to deliver the treatment to cells or to stop the delivery of treatment to the cells without mechanically affecting the cells.

Microfluidics devices have gained prominence for CNS cell calcium imaging due to the reduction in sample size, high temporal resolution, and single-cell resolution analysis^49^. The CAM-μTAS contributes to this effort by adding the spatial resolution factor to the analysis. During the administration of cytokine gradient to microglia, we observed that microglia respond to the treatment through a concentration gradient and several cells had more than one peak, as shown in Fig. 6, SI Fig. 4, and SI Video 2. This result shows that as the concentration of cytokines increases, cell activity also increases as indicated by multiple calcium transient peaks. This observation was only possible due to the delivery of cytokines through a concentration gradient. However, using the perfusion system, we could only observe an instant calcium dynamic at the application of the treatment and no further response from microglia. We plan to further investigate the cells that showed more than one calcium peak as the cytokine concentration increased in the microglia cell culture chamber. However, we believe that it is due to the heterogeneity in the microglia population.

The CAM-μTAS successfully allowed the monitoring of calcium dynamics by introducing various innovative features. However, the current device does not offer control over the distribution of cells in the chamber. This limitation can be addressed by designing cell blocks in the chamber that can hold single cells^47^. Additionally, the stability of flow in the microglia cell culture chamber can be improved by applying an external pressure to the microfluidic flow rectifier as described by Bavil, et al^43^. However, this technique comes at the cost of pumping efficiency.

The current device can be used for various applications including drug screening, cell-to-cell communication, and cell chemotaxis. For drug delivery, other brain cells, including neurons and astrocytes can be cultured in the microglia cell culture chamber and drugs can be delivered to the cell through the Quake valve following a concentration gradient and a response can be measured in real time. For cell-to-cell communication, different cell types can be cultured in the two chambers and the Quake valve can be actuated to allow the cells to communicate. Finally, the CAM-μTAS can be used to quantify cell chemotaxis. Here, a chemoattractant can be added in the cytokine chamber and a gradient can be generated through the Quake valve to allow the cells to migrate following the chemoattractant gradient. In conclusion, the addition of automation to microsystems promises a new era of precision, speed, and consistency in understanding and manipulating human cellular behavior, opening the doors for advancements in personalized medicine, disease modeling, and drug discovery.

## Conclusion

We have developed a calcium monitoring micro-total analysis system (CAM-μTAS) enabling the measurement of calcium dynamics in microglia following a cytokine treatment. Equipped with an automated fluid pump, microglia cell culture, and cytokine chamber separating Quake valve, this device enables programmed and metered fluidic manipulations with no human interaction. In this work, we have optimized and validated the CAM- μTAS for calcium imaging. We were able to quantify a location-dependent calcium dynamics response of microglia to a cytokine gradient. The device leverages the strength of both the lifting gate microvalve arrays and the Quake valve for cell loading and fluid processing including media change, cell incubation with calcium indicator, gradient formation, and cytokine delivery to the cells. The developed device is fabricated using a combination of both positive and negative photoresists to allow the incorporation of the two different valve systems. The CAM-μTAS introduces an innovative method in the fabrication of microfluidic pneumatic control systems.

### Supplementary information

SI

SI Video 1: Operation of the CAM-μTAS

SI Video 2: Microglia calcium dynamics in the CAM-uTAS
