# Supplementary material for: A programmable microfluidic platform to monitor calcium dynamics in microglia during inflammation
Source: Microsyst Nanoeng. 2024 Aug 1;10:106. doi: 10.1038/s41378-024-00733-1 (PMC11294448; doi:10.1038/s41378-024-00733-1)
Supplement: Supplementary file 1 — SI [file 41378_2024_733_MOESM1_ESM.docx]

**Supplementary Figures**


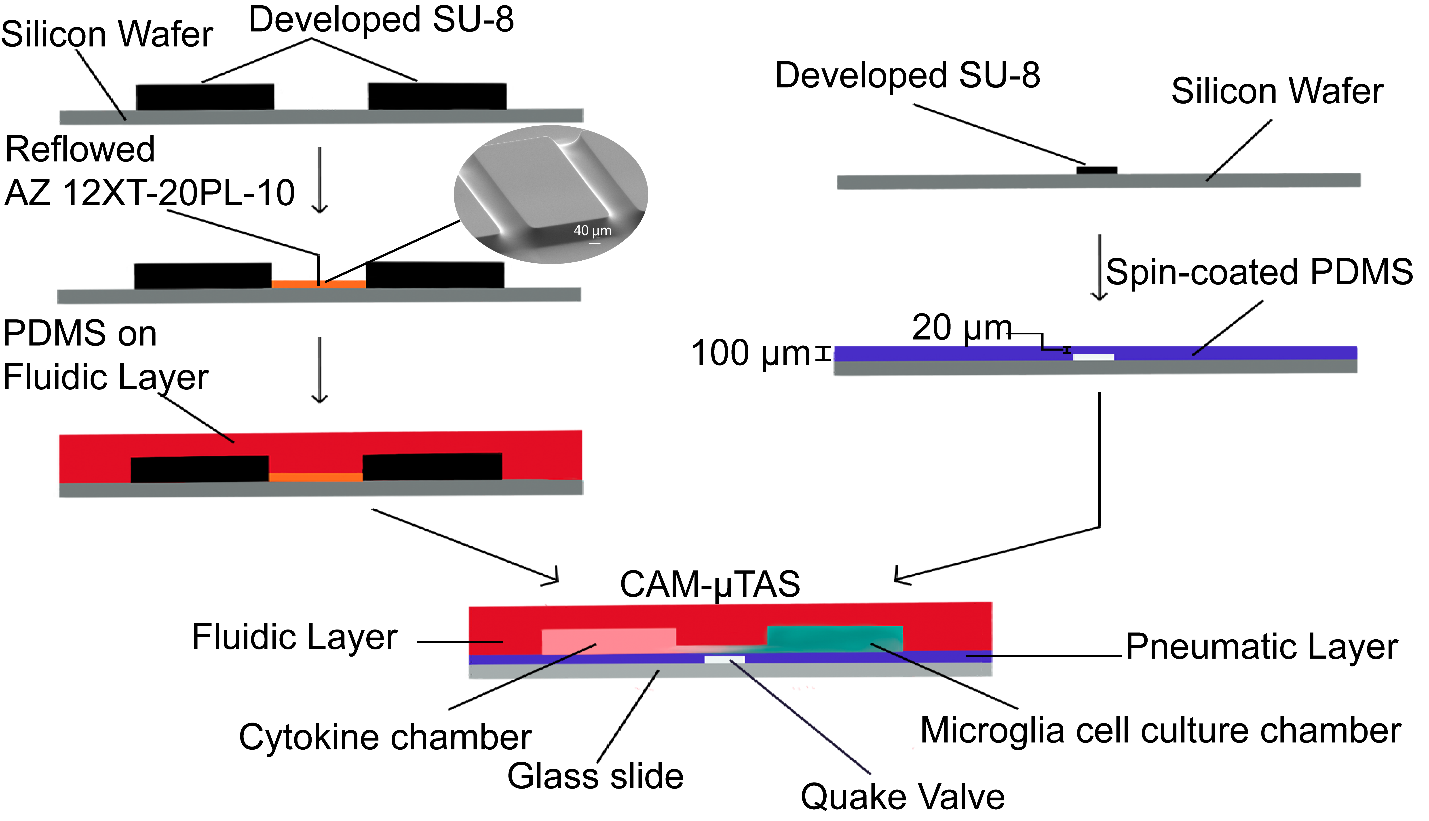


**SI Figure 1. Fabrication of the CAM-μTAS through an improved photolithography technique and soft lithography**. The photolithography included the use of Su-8 (negative photoresist) and AZ 12XT-20-PL-10 (positive photoresist). The later was reflowed to allow the Quake valve to have a semicircular roof. This method enables us to fabricate a system with both a negative and positive photoresist to incorporate both the Quake valve and lifting-gate valve into the CAM-μTAS.


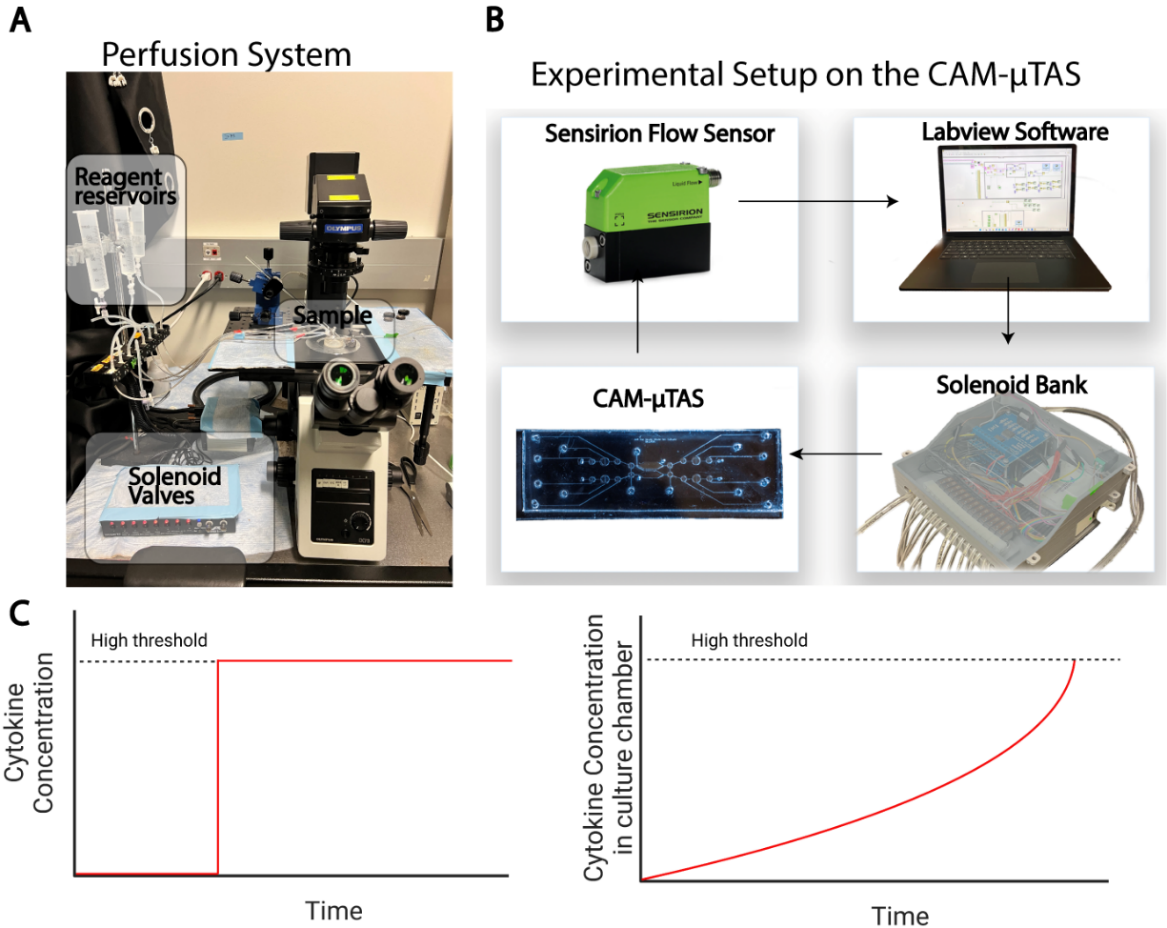


**SI Figure 2**. A) Experimental setup of the traditionally used perfusion system. The system uses 1 liter of reagents per experiment. B) Experimental setup of the CAM-μTAS includes the automated microfluidic device connected to solenoid valves which are connected to a microcontroller that allows the actuation of valves by a custom-made Labview software. Flow rate through the device is measured using a Sensirion flow sensor.

**Process of Actuation of Valves**

The CAM-μTAS has a series of lifting gate valves at the inlet (to the left of the microfluidic flow rectifier) to pump fluid in and a series of lifting gate valves at the outlet to pump out as shown in SI Figure 2. The device is controlled by actuating the valves in an open-close series as shown in SI Video 1. The waste liquid is collected in the outlet of the device.


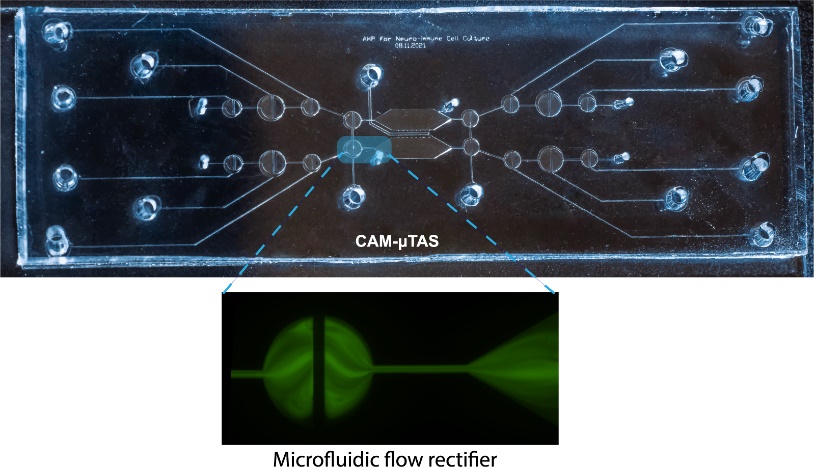


**SI Figure 3. Microfluidic flow rectifier.** We validated that the flow rectifying valve successfully linearized the flow around cells and eliminated backflow.


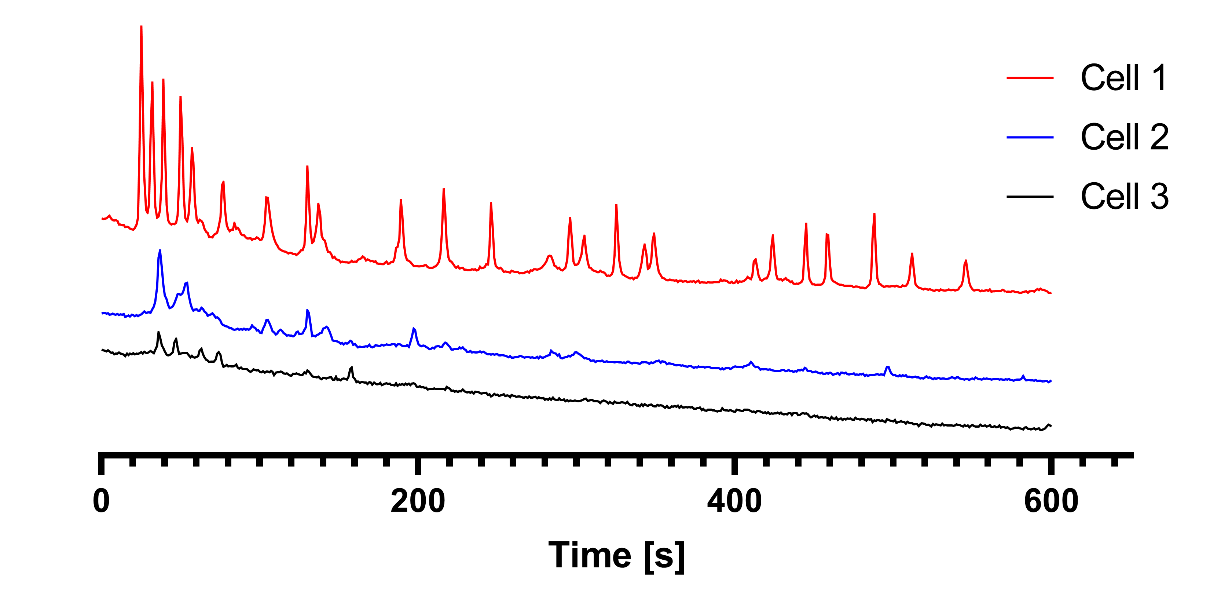


**SI Figure 4. Increase calcium responsiveness of cells with the concentration gradient.** Several cells displayed more than one calcium dynamics peak as the concentration increased in the chamber. This effect will be investigated to decipher why these cells had an increase in frequency of calcium dynamics response. Cells 1, 2 and 3 are located at 17.3 μm, 39 μm, 52 μm respectively, from the cytokine source .

**Supplementary Figures**

**SI Video 1.** CAM- -μTAS operation. From automated sample preparation to gradient control, the CAM -μTAS allowed us to change media, prepare samples, release cytokines + ATP into the cell culture chamber and wash the chamber for a subsequent experiment.

**SI Video 2**. After opening the Quake valve, cytokines + ATP activated microglia (BV2) following a concentration gradient with cells closer to the source activated first and the cells downstream activated progressively. The activation is shown through calcium dynamics.
